# Molecular Mechanisms of Synaptic Dysregulation in Fragile X Syndrome and Autism Spectrum Disorders

**DOI:** 10.3389/fnmol.2019.00051

**Published:** 2019-03-07

**Authors:** Michael Telias

**Affiliations:** Department of Molecular and Cell Biology, University of California, Berkeley, Berkeley, CA, United States

**Keywords:** synaptic plasticity, fragile X syndrome, autism spectrum disorders, mouse models, human pluripotent stem cells, human embryonic stem cells, human induced pluripotent stem cells

## Abstract

Fragile X syndrome (FXS) is the most common form of monogenic hereditary cognitive impairment. FXS patient exhibit a high comorbidity rate with autism spectrum disorders (ASDs). This makes FXS a model disease for understanding how synaptic dysregulation alters neuronal excitability, learning and memory, social behavior, and more. Since 1991, with the discovery of fragile X mental retardation 1 (FMR1) as the sole gene that is mutated in FXS, thousands of studies into the function of the gene and its encoded protein FMR1 protein (FMRP), have been conducted, yielding important information regarding the pathophysiology of the disease, as well as insight into basic synaptic mechanisms that control neuronal networking and circuitry. Among the most important, are molecular mechanisms directly involved in plasticity, including glutamate and γ-aminobutyric acid (GABA) receptors, which can control synaptic transmission and signal transduction, including short- and long-term plasticity. More recently, several novel mechanisms involving growth factors, enzymatic cascades and transcription factors (TFs), have been proposed to have the potential of explaining some of the synaptic dysregulation in FXS. In this review article, I summarize the main mechanisms proposed to underlie synaptic disruption in FXS and ASDs. I focus on studies conducted on the *Fmr1* knock-out (KO) mouse model and on FXS-human pluripotent stem cells (hPSCs), emphasizing the differences and even contradictions between mouse and human, whenever possible. As FXS and ASDs are both neurodevelopmental disorders that follow a specific time-course of disease progression, I highlight those studies focusing on the differential developmental regulation of synaptic abnormalities in these diseases.

## Introduction

Fragile X Syndrome (FXS) is the most prevalent form of inherited intellectual disability (Penagarikano et al., 2007). It is caused by a CGG triplet repeat expansion, in the 5′ UTR region of the fragile X mental retardation 1 (FMR1) human gene, located in the X chromosome (Verkerk et al., 1991). If this genomic region expands to more than 200 CGG-repeats, the promoter of FMR1 becomes hyper-methylated, resulting in the inactivation of the gene and the absence of its encoded protein: FMR1 protein (FMRP; O’Donnell and Warren, 2002; Mor-Shaked and Eiges, 2018). FMRP is an RNA-binding protein that plays important roles in regulation of translation as well as in other processes in the central nervous system (CNS; Bagni and Oostra, 2013; Fernández et al., 2013). It is estimated that FMRP has hundreds of mRNA and microRNA targets, making the study of FMRP’s role a very challenging field (Ascano et al., 2012; Pasciuto and Bagni, 2014). Accordingly, FXS pathology is complex too. Patients with FXS suffer from mild to severe cognitive impairment, epilepsy, auditory hyper-sensitivity, repetitive behavior, social withdrawal and other neurological symptoms, as well as other disorders outside of the CNS, like cartilage malformations and macroorchidism (Penagarikano et al., 2007; Kidd et al., 2014). Perhaps the most remarkable fact about FXS for the purposes of this review is a high comorbidity with autism spectrum disorders (ASDs): 50% of male FXS patients and 20% of female FXS patients are diagnosed with ASDs (Kaufmann et al., 2017). Therefore, studying FMRP’s role in the CNS can shed light not only on the etiology of FXS, but also on basic mechanisms shared with other neurodevelopmental disorders, while expanding current scientific understanding of synaptic plasticity and brain physiology (Telias and Ben-Yosef, 2014).

A critical feature of FXS pathophysiology is related to the timing of FMR1 expression and inactivation during human embryonic development (Colak et al., 2014). It is safe to assume that the full CGG-repeat expansion exists already in the zygote, since it can be detected as early as in human blastomeres (Malcov et al., 2007). Yet, despite the CGG-repeat expansion present, the FMRP protein is expressed and detected in FXS human embryonic tissue, at least up to the end of the first trimester of pregnancy (Willemsen et al., 2002; Mor-Shaked and Eiges, 2018). After this time-point, the FMR1 promoter becomes gradually hyper-methylated and the gene increasingly silenced. This means that, in human FXS embryos, early neurodevelopmental events such as the formation of the neural tube take place in the presence of FMRP; while FMRP absence probably affects late developmental stages, such as rapid neurogenesis from progenitors, migration and synaptogenesis, during the late phases of cortical and neo-cortical development (i.e., second and third trimesters). This is a critical feature to consider when assessing the mechanisms behind FXS pathology, but also when choosing research tools, because the CGG-repeat expansion at the FMR1 locus, and its ensuing developmentally-regulated disappearance of FMRP, is unique to humans and is not recapitulated in *Fmr1* knock-out (KO) mice (Eiges et al., 2007; Telias and Ben-Yosef, 2014).

In this review, I will summarize the main hypotheses and mechanistic models proposed to explain synaptic dysregulation in FXS and ASDs (see Table 1). All these hypotheses ultimately reflect the current state of knowledge regarding the role of FMRP in CNS neurons, during embryonic development and postnatal life. I will include studies conducted on the *Fmr1* KO mouse model, and emphasize how they compare to more recent research carried-out on human pluripotent stem cells (hPSCs), including human embryonic stem cells (hESCs) obtained from donated *in vitro* fertilization human blastocysts, and human induced pluripotent stem cells (hiPSCs) derived from somatic cells obtained from patients’ biopsies.

**Table 1:** Summary of mechanisms involved in Fragile X Syndrome (FXS) pathology.

Gene/Protein target	Mechanism	Developmental stage	Model	Reference
mGluR1, mGluR5	mGluRI-dependent LTD	Postnatal	Mouse	Bear et al. (2004)
AMPA and NMDA receptors	iGluR-dependent LTP	Postnatal	Mouse/hPSCs	Uzunova et al. (2014)
GABA-A (α1, α3)	ionotropic GABA-dependent inhibition	Postnatal and embryonic	Mouse/hPSCs	Braat and Kooy (2015)
GABA-B	metabotropic GABA-dependent inhibition	Postnatal and embryonic	Mouse/hPSCs	D’Hulst et al. (2009)
BK channels	abnormal intrinsic neuronal excitability	Postnatal	Mouse	Deng et al. (2011)
BDNF, TrkB	BDNF-dependent activity	Postnatal and embryonic	Mouse/hPSCs	Castrén and Castrén (2014)
Adenylyl Cyclase, others	cAMP-dependent signaling	Embryonic	Human Fetal Tissue	Kelley et al. (2007)
GSK3β	Wnt signaling, MAPK	Postnatal and embryonic	Mouse/hPSCs	Mines and Jope (2011)
SOX TFs	SOX-dependent neurogenesis	Embryonic	hPSCs	Telias et al. (2015b)

*Table 1 summarizes the mechanisms discussed in the review, found to be involved in the brain pathophysiology of FXS, according to the target gene or protein proposed. The table indicates the developmental stage and organism model used, as well as a representative article for each mechanism*.

## Chemical Synaptic Transmission

### Glutamate-Dependent Synaptic Transmission

Glutamate is the most prevalent excitatory neurotransmitter in the brain. Glutamate receptors (GluRs) are divided into two major families: ionotropic and metabotropic. Both types of GluRs are intrinsically involved in the activation of long-term potentiation (LTP) and long-term depression (LTD). Given that intellectual disability constitutes perhaps the most important aspect of FXS pathology and that synaptic plasticity is considered to be at the base of learning and memory, researchers raised the hypothesis that FMRP could be involved in the regulation of luRs. This approach could also uncover pharmacological targets for a possible therapy. Surprisingly, one of the first studies that assessed synaptic transmission in *Fmr1* KO mice showed no conclusive abnormalities (Godfraind et al., 1996). The affected mice showed normal acquisition of new behavior as compared to healthy counterparts, but difficulties during extinction of the learned behavior and the acquisition of a new one, suggesting impaired LTP. However, electrophysiological recordings showed no significant differences in LTP recordings carried out on hippocampal CA1 neurons in wild-type (WT) vs. *Fmr1* KO mice. The same study also showed that *Fmr1* expression is not affected by the induction of LTP in WT neurons, but it did not address the question whether LTP-responsive genes, including GluRs, are differentially expressed in WT as compared to *Fmr1* KO.

Breakthrough research by Huber et al. (2002) showed an increase in the expression of postsynaptic metabotropic GluR type-I (mGluRI) in *Fmr1* KO hippocampal neurons. mGluRs are G-protein coupled receptors that mediate slow response to glutamate. There are eight different mGluRs divided into three groups: mGluRI_(1,5)_, mGluRII_(2,3)_, and mGluRIII_(4,6,7,8)_ (Maj et al., 2016; Ribeiro et al., 2017). According to this hypothesis, mGluRI expression is negatively regulated by FMRP, and therefore, loss of FMRP results in an abnormal increase of mGluRI in *Fmr1* KO neurons, enhancing mGluR-dependent LTD. An increase in LTD, seemingly at the expense of LTP, would be consistent with intellectual disability and cognitive impairment, since these mechanisms have been shown to directly affect learning and memory. This fundamental result, the increase in mGluRI-dependent LTD in correlation with FMRP loss in mice, was later confirmed by many independent studies (Todd et al., 2003; Antar et al., 2004; Aschrafi et al., 2005; Desai et al., 2006; Huang et al., 2015) giving rise to the formulation of the “mGluR theory of FXS” (Bear et al., 2004; Bear, 2005), which will eventually rise to almost dominate the field of FXS research. Enhanced LTD mediated by mGluRs not only provides a possible biological explanation for the intellectual disability associated with FXS, but also provide highly-specific drug targets for a potential pharmacological treatment, or cure, of FXS (Sourial et al., 2013; Berry-Kravis, 2014; Gandhi et al., 2014).

Yet, the mGluR-based explanation of synaptic dysregulation in FXS has some weak points that need to be addressed. First, the molecular mechanism and the cascade of cellular events that lead from FMRP loss to mGluRI functional upregulation remains unresolved. Second, none of the molecular and physiological hallmarks of the “mGluR theory” have ever been conclusively confirmed in any human model for FXS or ASDs. Third, from a more neurodevelopmental perspective, the question of the timing of mGluRI hyperactivation remains open. If mGluRI hyperactivation is caused by FMRP downregulation, it is important to remember that, as mentioned before, the *Fmr1* mutation in KO mice does not recapitulate the much later timing of developmentally-regulated transcriptional inactivation, as observed in human embryos and hESCs (Telias et al., 2013; Telias and Ben-Yosef, 2014). One study tackled these questions directly, comparing developing neural progenitors obtained from WT and *Fmr1* KO mice and differentiated *in vitro* from healthy and FXS-hiPSCs (Achuta et al., 2017). By measuring Ca^2+^ responses to dihydroxyphenylglycine (DHPG, an agonist of mGluRI_(1,5)_), with or without the presence of 2-methyl-6-(phenylethynyl)pyridine (MPEP, a selective mGluR_5_ antagonist), they could measure the contribution of mGluR5 to mGluRI-hyperactivation. In murine *Fmr1* KO cells, the presence of MPEP did not significantly change Ca^2+^ responses to DHPG, while in human FXS-hiPSCs derived neural progenitors, blocking mGluR_5_ tripled the number of cells positively responding to DHPG. These results, even though not yet confirmed by other studies, seem to indicate that the alterations in mGluR-dependent signaling found in mice do not necessarily correlate to the pathophysiology found in human models. A clinical trial by Novartis with mavoglurant (AFQ056), an mGluR5 antagonist, was discontinued after it failed to show improvement over placebo in FXS patients (NCT01482143). Another study, analyzing the data from two separate phase-IIb trials of mavoglurant administration to fully-methylated FXS patients, reached the conclusion that neither of the studies achieved the primary efficacy end-point of improvement on behavioral symptoms (Berry-Kravis et al., 2016).

Finally, it is worth mentioning that mGluRI activation is found to be abnormal in other neurodevelopmental disorders, in which FMR1 and FMRP are not mutated in any way, and are normally expressed in the CNS. For example, in one study conducted in the cerebellar inner granular layer of Neuroligin-3 KO mice (with normal expression of *Fmr1*), an increase in the expression of mGluR1α was found, together with an increase in the phosphorylation of the GluA2 subunit of ionotropic GluRs (iGluRs) and the occlusion of mGluR-LTD upon treatment with DHPG (Baudouin et al., 2012). Another model for ASDs, known as the BTBR mouse (Meyza and Blanchard, 2017), was used to show that behavioral deficits associated with ASDs in these mice, are reversed by treatment with MPEP (Seese et al., 2014). Mice displaying haploinsufficiency of synaptic GTPase-activating protein (*Syngap*^+/−^) are another monogenic model for ASDs (Jeyabalan and Clement, 2016). One study directly compared hippocampal physiology in *Fmr1* KO and *Syngap*^+/−^ mice, finding that they both show the same elevated mGluRI-dependent LTD (Barnes et al., 2015). In summary, increased mGluRI signaling in *Fmr1* KO mice: (1) is caused by a molecular mechanism that has not been yet fully elucidated; (2) it has never been shown to be true in human neuronal tissue; *in vivo* or *in vitro*; (3) it has failed to provide an effective drug target to ameliorate FXS; and (4) it has been shown to exist in many other mouse models of intellectual disability and ASDs, regardless of the *Fmr1* KO mutation. Taken together these findings suggest that increased mGluRI-dependent LTD is a common consequence of intellectual disability and not the cause of it.

iGluRs include α-amino-3-hydroxy-5-methyl-4-isoxazolepropionic acid (AMPA), N-methyl-D-aspartate (NMDA) and kainate receptors. Research into the possible involvement of iGluRs in FXS and ASDs synaptic pathophysiology, has been less prevalent as compared to the study of mGluRs (Uzunova et al., 2014). As mentioned before, in perhaps the first of such studies, Godfraind et al. (1996) did not find evidence for altered LTP in the hippocampi of adult *Fmr1* KO mice. However, since then, new research has emerged challenging this concept, but also providing somewhat conflicting results. One study showed reduced LTP and decreased levels of GluR1-containing AMPA receptors in the cortex of *Fmr1* KO mice, but normal LTP in the hippocampus and normal levels of GluR1 in both hippocampus and cerebellum (Li et al., 2002). In contrast, another study showed reduced LTP in hippocampal CA1 neurons of *Fmr1* KO mice, associated with reduced delivery of GluR1-containing AMPA receptors to the active synapse, but without a change in GluR1 expression (Hu et al., 2008). The same study also showed that enhancement of the Ras-PI3K signaling pathway rescues LTP in these mice, but the exact mechanism linking loss of FMRP to this pathway remains unknown. In this context, one study explored the question whether changes in LTP associated with FXS are developmentally regulated (Pilpel et al., 2009). They found that CA1 hippocampal neurons of 2-weeks old *Fmr1* KO mice show down-regulation of AMPA receptors and up-regulation of NMDA, significantly changing the AMPA to NMDA ratio, resulting in enhanced NMDA-dependent LTP. Most interestingly, they found that by age 6–7 weeks, these abnormalities in synaptic plasticity disappeared, suggesting the existence of a critical developmental period in which loss of FMRP could account for impaired plasticity. More recent studies further show the complexity of FXS-associated synaptic deficiencies and their time-dependency. For example, one study showed increased mGluR-dependent LTD in *Fmr1* KO hippocampal neurons upon NMDA receptor blockade, as expected (Toft et al., 2016). However, while this was the case at P30, the same was not found at P60, indicating that enhanced mGluR-LTD in *Fmr1* KO mice is NMDA-dependent and developmentally regulated.

All of the aforementioned studies analyzing the possible role of iGluRs in synaptic dysregulation in FXS were conducted using the mouse model for FXS, and examining mostly hippocampal CA1 neurons. Far less studies have been conducted in other brain regions, and even less in other FXS models. One recent study in *Fmr1* KO mice focused on the Mossy fiber pathway which innervates CA3 hippocampal neurons, showing increased excitatory postsynaptic potentials coupled with enhanced AMPA receptor activation (Scharkowski et al., 2018). A study conducted on 8-weeks old *Fmr1* KO rats, showed deficient AMPA receptors-mediated responses as compared to WT in hippocampal CA3-CA1 synapses (Tian et al., 2017). Interestingly, this study also shows a decrease in both LTP and LTD in *Fmr1* KO rats as compared to WT, and an increase in DHPG-induced LTD in *Fmr1* KO, which was independent of protein synthesis. Finally, a recent study conducted on human neurons derived from FXS-hiPSCs shows, for the first time, functional changes in AMPA receptors in a human model (Achuta et al., 2018). This study analyzed the differential expression and activation of iGluRs during the process of *in vitro* differentiation, which can be correlated, to some extent, to human embryonic development. The results show decreased GluR2 expression, and increased expression of Ca^2+^-permeable AMPA receptors, in human FX neurons as compared to non-mutated controls. The number of cells co-expressing AMPA and NMDA was higher in FX neurons, too. However, in striking opposition to most studies conducted on *Fmr1* KO mice, there was no significant difference in the fraction of DHPG-responsive cells in FX vs. control. These findings exemplify how disappearance of FMRP has different effects on human neurons as compared to rodent counterparts, and can also hint at a critical (and maybe overlooked) difference between the role of FMRP during embryonic development and early life on one hand, and during adulthood on the other.

#### Gamma-Aminobutyric Acid-Dependent Synaptic Transmission

The major inhibitor neurotransmitter in the brain is an enzymatic product of glutamate break-down, gamma-(γ)-aminobutyric acid (GABA). Two major families of GABA receptors exist: GABA_A_ (ionotropic) and GABA_B_ (metabotropic; Fritschy and Panzanelli, 2014; Mele et al., 2016). GABA_A_ receptors are expressed in the whole CNS, and their activation is coupled with a fast increase in chloride conductance and hyperpolarization of the postsynaptic neuron, inhibiting neuronal activity. GABA_B_ receptors have a similar inhibitory function, but through slower G-protein mediated activation of K^+^ channels. GABA_A_ receptors are pentameric, and composed of combinations of several different subunits. Some of these subunits also include several different isoforms, which makes the study of GABA_A_ receptors structure and composition a specially challenging field. GABA_B_ receptors are similar in structure to mGluRs, and are divided into two subtypes that assemble as heterodimers. Two key symptoms, present in both FXS and autism, are hyperexcitability and hypersensitivity, which could be caused by reduced GABA-mediated inhibition. If this hypothesis is true, then FXS neurons should exhibit decreased GABA receptors expression, reduced GABA secretion, or both, reducing inhibition and therefore increasing uncontrolled excitation.

And indeed, both GABA_A_ and GABA_B_ receptors have been found to be involved in FXS and ASDs pathology, during embryonic development and in adulthood. Several studies have shown a reduction in the mRNA expression of several GABA_A_ receptor subunits in correlation with the loss of FMRP (D’Hulst and Kooy, 2007; Paluszkiewicz et al., 2011; Braat and Kooy, 2015; Braat et al., 2015), but the mechanism of this effect remains unclear, especially since FMRP is known as a negative regulator of translation. Seminal work by D’Hulst and Kooy (2007) showed that, in the cortex of *Fmr1* KO mice but not in their cerebellum, mRNA levels of eight different GABA_A_ subunits displayed a down-regulation of 35%–50%, including most prominently the δ subunit, as well as α1, α3, α4, β1, β2, γ1 and γ2 (D’Hulst et al., 2006). However, the authors did not provide any insight on whether these changes in GABA_A_ receptor subunit mRNA expression are developmentally regulated. This is important, since GABA_A_ receptor subunit expression is itself developmentally-regulated (Luján et al., 2005). Many follow-up studies have confirmed the fundamental results obtained by D’Hulst et al. (2006) consistently showing a reduction in GABA_A_ subunits expression in different parts of the brain, including the hippocampus and the amygdala, concomitant with the expected alterations in GABAergic synaptic transmission, such as reduced inhibitory postsynaptic potentials and currents (Olmos-Serrano et al., 2010; Sabanov et al., 2017; Zhang et al., 2017). However, while the literature is rich in reports confirming the “GABAergic theory of FXS” in esoteric models of the disease, such as zebrafish and *Drosophila*, the same literature to-date includes only one single report attempting to test this hypothesis in FXS human patients (D’Hulst et al., 2015). This study, using positron emission tomography (PET) to map GABA_A_ receptor availability in 10 FXS patients, found an average reduction throughout the brain of only 10%, far from the almost 50% reduction expected from mouse studies, with the thalamus being the brain region showing the most significant reduction (17% as compared to control subjects). Since the thalamus is not a brain area typically associated with learning, memory and complex social behavior, the results shown in this study actually raise more questions than they solve. In spite the lack of confirmation in humans and contradicting results, clinical trials with different drugs aimed at enhancing GABA_A_ signaling were approved and conducted (Erickson et al., 2011; Braat and Kooy, 2015), failing to provide the expected clinical improvement.

As for GABA_B_ receptors, it has also been shown that their expression is reduced in the forebrain of adult *Fmr1* KO mice (D’Hulst et al., 2009). This study proposes that hyperexcitability in FXS is caused by decreased GABA_B_-mediated attenuation of glutamate secretion at presynaptic terminals. It was subsequently reported that treating adult (8–12 weeks old) autistic mice with R-Baclofen, a GABA_B_ specific agonist, effectively reversed social deficits and reduced repetitive behavior (Silverman et al., 2015). More recent data seems to support the idea that of a specific FXS-associated deficit in GABA_B_ receptors subunit expression in presynaptic terminals, which could lead to excess secretion of glutamate in the hippocampi of 5-week-old *Fmr1* KO mice (Kang et al., 2017). Here too, the researchers made the effort to confirm, in post-mortem human brain tissue, the observations collected from the mouse model. The results, although not significant, showed a trend toward human validation of the mouse results: a decrease in the protein levels of a few GABA_B_ subunits. However, and most importantly, this study also shows that treatment with R-Baclofen does not rescue abnormalities in synaptic activity characterizing *Fmr1* KO neurons. Currently, one human clinical trial (NCT01013480), testing the efficacy of R-Baclofen as a candidate drug for FXS treatment, has reported unsuccessful results.

The only other published study so far, aimed at testing the “GABAergic theory of FXS” in human neurons is our own (Telias et al., 2016). In it, FXS human neurons were differentiated *in vitro* from three different lines of FXS-hESCs, all affected with the naturally occurring >200 CGG expansion mutation. By puffing GABA during whole-cell patch-clamp recordings, we showed that developing human neurons display either a mature response (bicuculine-sensitivity, no current desensitization), and an immature response (bicuculine-insensitive, fast and lasting desensitization). While 60% of the WT neurons tested were classified as immature and 40% mature, ~90% of the developing FX human neurons showed immature responses. Furthermore, the transcriptional levels of the GABA_A_ β_2_-subunit were dramatically reduced in FX human neurons, in accordance to some of the findings in *Fmr1* KO mice. However, the expression of δ was similar in FX and WT, and the expression of α_2_ was increased in FX neurons, two results that directly contradict the evidence obtained using the *Fmr1* KO mouse model, which shows significant reduction in δ and no effect on α2 (D’Hulst et al., 2006). None of the cells analyzed in our study, FX or WT, mature or immature, responded to baclofen, demonstrating a lack of functional GABA_B_ expression during this developmental stage, regardless of FMR1/FMRP expression.

As mentioned before, GABAergic synaptic transmission and chloride gradient regulation, play a crucial role in brain development, and might also explain the developmental aspects of FXS and ASDs. During early development of the CNS, GABA_A_ receptors are key players in an excitatory-to-inhibitory developmental switch (Ben-Ari, 2014). Impairment of this developmental switch has been proposed as a pathological molecular mechanism shared by several neurodevelopmental disorders (Ben-Ari, 2017). And indeed, in *Fmr1* KO mice, it has been found that this developmental switch is delayed, in correlation with a significant increase in the expression of the neuronal chloride transporter NKCC1 (He et al., 2014). Using tissue sections containing somatosensory cortex from P5-15 *Fmr1* KO and WT mice, this study demonstrated delayed maturation of Cl^−^ currents in the affected mice, in correlation to protein expression levels measured by Western blot. According to this model, during human embryonic development, FXS-neurons remain depolarized for longer, presumably delaying their maturation and affecting synaptogenesis, during a critical period in neurogenesis. In the follow up to this study, the researchers show that inhibition of NKCC1 in *Fmr1* KO during this critical period, corrects the Cl^−^ imbalance and rescues the phenotype *in vivo* (He et al., 2018). This is a powerful model, that can both, explain many of the symptoms associated with FXS, as well as providing a pathway for a possible treatment.

## Neuronal Excitability

Beyond aberrant expression and function of neurotransmitter receptors, other cellular and molecular neuronal mechanisms could be disrupted in FXS and ASDs, including abnormal excitability, neurotransmitter release and synaptogenesis. Studying the effect of FMRP loss on basic neuronal electrical properties, such as action potential (AP) firing, membrane resistance, ion channel expression and current conductance; as well as release probability and dynamics, and vesicle composition; could prove important in understanding the pathophysiology of FXS and ASDs. One of the first and more interesting studies to tackle this question, made used of an unorthodox FXS mouse model: a mouse displaying mosaic expression of *Fmr1* (as it is in FXS females), including a reporter gene (GFP), to allow discrimination between cells based on whether they express FMRP, or not (Hanson and Madison, 2007). Electrophysiological recordings from coupled cells (in four possible combinations), from CA3 pyramidal neurons, showed a reduction in the proportion of active synaptic connections from 70% when the presynaptic cell expressed *Fmr1*, to 40% when the presynaptic neuron was *Fmr1* KO, while the average amplitude of excitatory postsynaptic currents did not change in correlation to FMRP expression. This seems to indicate that FMRP absence results in reduced synaptogenesis, but within those connections that successfully developed, the postsynaptic response seems to be unaffected. Lack of altered postsynaptic activity could explain why the phenotype of the mosaic FXS female is much milder than that found in the majority of FXS males. Importantly, these recordings took place during the critical period at age P5-6, but were not compared to recordings carried out after it. Another study seems to independently support this idea, by analyzing the proteasome expression profile of isolated synaptic membranes from P14 WT and *Fmr1* KO hippocampi (Klemmer et al., 2011). This screening showed that lack of FMRP affects several presynaptic proteins, including a reduction of ~40% in the expression of β-Catenin [*Ctnnb1*, see glycogen synthase kinase 3 beta (“GSK3β”) below], and an increase of ~25%–40% in the expression of Synapsin (*Syn1*), and Synaptophysin (*Syp*), which are involved in regulation of synaptic vesicle release and the formation of new synaptic connections. The study also shows that FMRP loss is associated with a reduced density of vesicles per cluster and a higher proportion of docked vesicles, indicating reduced synaptic activity, in line with the work of Hanson and Madison (2007).

Two groundbreaking studies were published by the Klyachko lab (Deng et al., 2011, 2013), in which they reported important electrophysiological abnormalities directly affecting short-term plasticity in hippocampal pyramidal neurons of P15-25 *Fmr1* KO mice. They found that absence of FMRP in presynaptic neurons is correlated with enhanced responses to high-frequency stimuli and reduced short-term plasticity; as well as an increase in Ca^2+^ influx, synaptic vesicle recycling, and vesicle pool size. They also found that these FXS-enhanced excitatory responses to high-frequency stimuli were independent of GABAergic transmission. FMRP was found to regulate neurotransmitter release by modulating AP duration. This work demonstrated a critical role for FMRP through direct interaction with the regulatory β4 subunit of big potassium channels (BK). This protein-protein interaction was found to be translation-independent, expanding the spectrum of FMRP functions beyond negative regulation of translation through mRNA sequestration, and providing a new model to explain FXS and ASDs’ molecular pathophysiology.

Other important ion channels, regulating spiking and neurotransmitter release, might be affected in FXS. For example, Ferron et al. (2014) showed an increase in the function, density, and expression of N-type voltage-gated Ca^2+^-channels, in cultured E18 rat dorsal root ganglion cells, following shRNA-mediated knock-down of *Fmr1* expression. This study further demonstrated a direct protein-protein interaction between FMRP and Ca_v_2.2, somewhat similar to that found between FMRP and BK channels. Our own work was the first to report presynaptic abnormalities in human FX neurons differentiated *in vitro* from FXS-hESCs (Telias et al., 2013, 2015a). First, we found FXS-associated impairments in both neurogenesis and synaptogenesis, including the inability of human FX neurons to fire trains of consecutive APs. We also found specific abnormalities in inward and outward ionic currents and synaptic vesicle release dynamics, associated with FMRP loss. We demonstrated that impairments in early synaptogenesis associated with FXS display a presynaptic component, by co-culturing early human neurons with adult, fully-developed, rat neurons: in regular cultures human FX neurons showed poor spontaneous synaptic activity, but when these cells were differentially labeled and co-cultured with normal adult rat neurons, normal postsynaptic activity was restored. The study of FMRP’s effect on the expression of ion channels through protein-protein interactions is a fascinating new prospect in the research of FXS. However, it is still unclear how these new discoveries can be implemented in a clinical set-up to device therapeutic strategies.

## Cell Signaling, Growth Factors and Gene Expression

Synaptic transmission affects downstream cell signaling, and it is itself affected by upstream gene regulation events. Therefore, molecular interactions occurring “far away” from the synaptic domain, can have a direct impact on synaptic communication. In order to explore the involvement of growth factors, enzymes and transcription factors (TFs) in FXS and ASDs, we need first to assess whether these mechanisms are important in embryonic neurodevelopment, in adult neurogenesis, or in both. Molecular players affecting embryonic neurogenesis and synaptogenesis can be helpful in explaining how the impairments observed in FXS and ASDs patients came to be. Mechanisms affecting adult neurogenesis can help answer the question whether these impairments are reversible or not. Finally, mechanisms affecting both, embryonic and adult neurogenesis and synaptogenesis, could prove essential in designing therapeutic approaches to ameliorate or even cure neurodevelopmental disorders. Hypothetically speaking, it could be possible 1 day to treat FXS and autism patient through gene therapy as soon as they are born or even *in utero*, with the hopes of correcting any synaptic abnormalities before the brain is fully developed. Another hypothetical treatment for FXS and ASDs might 1 day be the implant of unmutated adult neural stem cells (aNSCs), that can re-populate the hippocampus and other areas with properly functioning neurons (Telias and Ben-Yosef, 2015), in the same way as today mutated hematopoietic stem cells are replaced with healthy ones. However, no strategy currently exists to re-populate the brain with healthy neurons, that can be directed to recapitulate proper neuronal wiring. In any case, for any therapeutic approach to work, elucidation of mechanistic abnormalities must be achieved first. Next, I summarize some of the most interesting studied mechanisms known to affect embryonic and adult neurogenesis, that have also been shown to be impaired in FXS and ASDs.

### BDNF

Brain-derived neurotrophic factor (BDNF) is a pivotal neuronal growth factor, involved in embryonic and adult neurogenesis (Park and Poo, 2013; Vilar and Mira, 2016), it is secreted from neurons and other cells in an activity-dependent manner, coupled to firing of APs, exerting its effect through both paracrine and autocrine routes, and binding of two different receptors: TrkB and p75. BDNF expression and secretion regulates synaptic plasticity in the hippocampus (Leal et al., 2015), is linked to AMPA and NMDA-mediated increase in Ca^2+^ influx, and can trigger PKC-mediated inhibition of GABAergic postsynaptic signaling (Henneberger et al., 2002; Slack et al., 2004). Critical roles have been found for BDNF in neurogenesis, dendritogenesis and synaptogenesis, while abnormalities in BDNF activity and specific polymorphisms in its sequence are associated with several diseases, including neurological and psychiatric disorders such as schizophrenia, epilepsy and drug addiction (Nagahara and Tuszynski, 2011). Impairments in BDNF-mediated signaling have also been associated with autism (Connolly et al., 2006; Correia et al., 2010).

The possible role of BDNF dysregulation in FXS and autism has been pioneered by the Castrén lab (Castrén and Castrén, 2014). First, they found that *Fmr1* mRNA levels are reduced *in vitro* in cultured mouse hippocampal neurons, when these cells are incubated with BDNF (Castrén et al., 2002). This effect was BDNF-specific and could not be mimicked by another neurotrophin, NT-3. Moreover, they showed *in vivo* that transgenic mice, overexpressing TrkB, display reduced hippocampal *Fmr1* and FMRP levels. However, they found no change in BDNF or TrkB expression in *Fmr1* KO mice. Later work uncovered a more complex picture regarding the involvement of BDNF in FXS (Louhivuori et al., 2011). BDNF protein levels were found to be increased in the hippocampus, and decreased in the cortex, of *Fmr1* KO mice, indicating that the possible role of BDNF in FXS is region-specific. However, both cortical and hippocampal neurons from *Fmr1* KO mice showed increased dendritic localization of BDNF-mRNA, in basal conditions and upon pharmacological induction of seizures. These observations are consistent with the role of FMRP as a local-dendritic negative regulator of mRNA translation. Furthermore, this work also suggested that abnormalities in BDNF-TrkB signaling might explain abnormal differentiation and migration of *Fmr1* KO neural precursor cells. Subsequently, *Bdnf* KO mice were cross-bred with *Fmr1* KO mice, creating double-mutant FXS animals with reduced BDNF expression (Uutela et al., 2012). In these double mutant mice, hippocampus-dependent learning and memory (i.e., Morris water maze) was shown to be negatively affected, as compared to WT, *Bdnf* KO, and *Fmr1* KO mice. However, other behaviors were positively impacted in the double-mutant mice, including locomotor activity and startle responses to loud noises, showing again region-specificity for BDNF role in FXS. They also explored the role of fluoxetine (Prozac), a selective serotonin reuptake inhibitor, in *Fmr1* KO mice (Uutela et al., 2014). Chronic administration of fluoxetine was previously shown to increase the expression of BDNF-induced LTP-associated genes in a brain region-specific pattern in mice (Alme et al., 2007), and to increase BDNF serum levels in humans (Liu et al., 2014). *Fmr1* KO mice treated with fluoxetine showed mixed results. Anxiety-like behavior was reduced in *Fmr1* KO mice, but also in WT counterparts. Locomotor hyperactivity was corrected in *Fmr1* KO mice by fluoxetine, but exploratory activity was abnormally high following treatment. At the cellular level, they found that fluoxetine significantly increased hippocampal cell proliferation in WT, but not in *Fmr1* KO mice, consistent with the idea that FMRP loss dysregulates BDNF signaling, and indicating that this effect might not be reversible. Finally, a more recent study showed the potential clinical significance of BDNF as therapeutic target in FXS (Nomura et al., 2017). In this study, the physiological maturation of fast-spiking interneurons in the sensory cortex of neonates was found to be delayed in *Fmr1* KO mice, and rescued by chronic delivery of a TrkB agonist. The study shows how a temporary decrease in TrkB activation during a critical period of synaptogenesis and circuit formation could be responsible for many of the deficits observed in FXS, and how restoration of TrkB activation could reverse these effects. However, the study did not show any data indicating that adult *Fmr1* KO mice show signs of behavioral rescue, if they are treated with a TrkB agonist as neonates. Yet, it strengthens the idea that BDNF role in FXS could be pivotal to the development of a successful treatment or cure.

### cAMP

The cyclic adenosine monophosphate (cAMP) signaling pathway is virtually ubiquitous in mammalian tissues. In neurons, cAMP signaling through the TF cAMP-responsive element binding-protein (CREB), has been involved in regulation of synaptic transmission, plasticity, and neurogenesis (Nicol and Gaspar, 2014; Ortega-Martínez, 2015). G-protein coupled receptors activate the membrane-bound enzyme adenylyl cyclase, which synthesizes cAMP from ATP, activating CREB. cAMP itself also serves as a ligand to many enzymes, most importantly PKA and cyclic nucleotide-gated channels such as HCN, which are crucial for membrane depolarization and neuronal spiking (Waltereit and Weller, 2003; Seino and Shibasaki, 2005; Biel and Michalakis, 2009; Baudry et al., 2015).

Several studies point toward possible abnormalities in the cAMP pathway in FXS and ASDs. Early studies showed a reduced production of cAMP in human FXS platelets, and that overexpression of FMR1 in human neural cells *in vitro* results in increased cAMP levels (Berry-Kravis and Sklena, 1993; Berry-Kravis et al., 1995; Berry-Kravis and Ciurlionis, 1998). These results were later corroborated in the same human *in vitro* cellular systems, as well as in *Fmr1* KO mice (Kelley et al., 2007), suggesting the existence of a conserved role for an “FMRP-cAMP pathway.” Importantly, altered cAMP signaling has also been correlated with autism (Kelley et al., 2008). Based on this body of evidence, cAMP became the target of a new therapeutic strategy, in which the goal is to increase cAMP levels in FXS patients. One study used Rolipram, an inhibitor of PDE-4, the cAMP-degrading phosphodiesterase, to increase the levels of cAMP in *ex vivo* acute hippocampal slices of *Fmr1* KO mice (Choi et al., 2015). Acute treatment with Rolipram resulted in a decrease in mGluR-dependent LTD, rescuing the *Fmr1* KO phenotype. A subsequent study raised the hypothesis that other drug candidates for FXS and ASDs treatment, including antagonists against mGluRs and GSK3β, also work by increasing cAMP levels (Choi et al., 2016). The method used to quantify the effect of these drugs was semi-quantitative western blot ratios between target proteins in their phosphorylated state vs. non-phosphorylated, and the ratio of the target protein to Tubulin, in hippocampal lysates of WT and *Fmr1* KO mice. Also, ERK activation was measured in lymphocytes. However, and most importantly, no electrophysiological or behavioral data from mice was provided, and it was not shown whether these treatments indeed increase cAMP concentration *in vivo* in mice. Moreover, many important questions remain open, it is yet not clear how does FMRP increase cAMP levels, and how exactly reduced levels of cAMP cause enhanced mGluR-dependent LTD.

### GSK3β

The Wnt/GSK3β pathway is essential for both embryonic and adult neurogenesis (Toledo et al., 2008; Kuwabara et al., 2009; Gage and Temple, 2013; Bengoa-Vergniory and Kypta, 2015). Wnt is the collective name of a family of secreted protein ligands that bind to membrane-bound receptors in the target cell, activating a signaling cascade. This cascade hits a main “cross-roads” when it activates cytoplasmic GSK3β, phosphorylating it. Phospho-GSK3β activates and inhibits several different pathways, including MAP-kinase, Cyclin and Akt, as well as β-Catenin as part of the canonical Wnt signaling pathway, resulting in complex gene regulation events that are yet not fully understood (Hur and Zhou, 2010; Seira and Del Río, 2014).

In FXS, it has been shown that GSK3β is elevated in the hippocampi of *Fmr1* KO mice, leading to abnormal adult hippocampal neurogenesis (Portis et al., 2012). Ablation of FMRP in aNSCs caused an increase in GSK3β protein levels, causing a phosphorylation-dependent decrease in β-Catenin activation and an increase in β-Catenin degradation, impairing neuronal differentiation (Luo et al., 2010), while pharmacological inhibition of GSK3β led to rescue of impaired neurogenesis *in vivo* (Guo et al., 2012). Moreover, several studies showed that lithium, an FDA-approved drug that inhibits GSK3β activity, alone or in conjunction with mGluR5 antagonists, mitigated many of the symptoms of FXS in *Fmr1* KO mice, from the cellular level to behavior associated with FXS and ASDs (Min et al., 2009; Yuskaitis et al., 2010; Mines and Jope, 2011).

However, many different lines of evidence argue against the idea of GSK3β as a legitimate target for FXS treatment. First, a study in which mice were fed with control or lithium-containing chow, reported that a lithium-rich diet failed to significantly alter the phosphorylated-to-non-phosphorylated GSK3β ratio (p-GSK3β/GSK3β), in the hippocampi of WT or *Fmr1* KO mice (Choi et al., 2016). They also demonstrated that even under a normal control diet, WT and *Fmr1* KO mice display non-significant difference in their p-GSK3β/GSK3β ratio. Second, it is possible that hyperactivation of GSK3β in *Fmr1* KO mice is rodent-specific. When FXS-hESCs were differentiated into human neural progenitor cells, no significant change in the transcription or translation of GSK3β or β-Catenin was found (Telias et al., 2015b). Overexpression of FMR1 in FXS-cells and siRNA-mediated knock-down of FMR1 expression in WT counterparts, failed to alter the levels of GSK3β or β-Catenin. Pharmacological inhibition of either GSK3β or β-Catenin affected neuronal differentiation of WT and FXS-human neural precursor cells similarly, regardless of FMRP levels. Finally, and most importantly, in the first clinical trial for lithium treatment of FXS patients, in which a placebo control was not included, the results sadly fell short of the stipulated clinical goals, while improvements were observed only in minor aspects of FXS pathology (Berry-Kravis et al., 2008; Liu and Smith, 2014). Currently, 10 years later, there are no active clinical trials involving lithium treatment for FXS or autism.

### Transcription Factors

During embryonic and adult neurogenesis, all gene expression must be triggered by the timely and correct activation of TFs. Different groups of TFs dictate the embryonic and adult neurogenesis of neural progenitor cells. One such group of TFs is known as the SOX superfamily, which in humans includes 20 different genes arranged in nine different sub-groups (Wegner and Stolt, 2005; Kiefer, 2007). For example, activation of SOX2 is critical for the development of neural progenitor cells, but also for maintaining their specific identity, meaning that for the progenitor to progress into a fully differentiated neuron, SOX2 expression should be de-activated. Our own study has shown a causative link between disappearance of FMRP and a significant increase in SOX2 levels in human FXS-neural precursor cells derived from FXS-hESCs (Telias et al., 2015b), coupled with poor neurogenesis in FX cells as compared to WT counterparts. Another member of the same family, SOX9, has been shown to induce neural crest development, gliogenesis and chondrogenesis (Marshall and Harley, 2000; Lee and Saint-Jeannet, 2011; Lefebvre and Dvir-Ginzberg, 2017). Several studies have pointed toward abnormal gliogenesis as a contributing factor in FXS and autism pathophysiology (Cheng et al., 2012), and loose, incompletely formed cartilage is a hallmark of non-neuronal symptoms of FXS, as well as craniofacial abnormalities (Penagarikano et al., 2007). We found that loss of FMRP results in a significant decrease in SOX9 expression, which is reversed by overexpressing FMR1 in human FXS neural progenitor cells and mimicked in WT cells by inhibiting FMR1 expression.

Other studies have also shown abnormal expression of TFs in human FX cells. We reported on abnormal expression of PAX6 and NOTCH1 during neural differentiation of FXS-hESCs (Telias et al., 2013). Halevy et al. (2015) studied the expression of repressor element-1 silencing TF (REST) in undifferentiated FXS-hiPSCs and their derived neurons. They found that undifferentiated FXS-hiPSCs have reduced expression of REST as compared to WT counterparts, but differentiated FXS-neurons display increased REST expression. REST is a negative regulator of neuronal development, it is expressed in early neural lineages but inactivated in mature neurons, similar to SOX2. Therefore, data from independent studies carried out on different *in vitro* human models of FXS (Halevy et al., 2015; Telias et al., 2015b), seem to indicate that FMRP loss results in a failure to inactivate the expression of negative regulators of neurogenesis, an idea that is consistent with the role of FMRP as a negative regulator of translation, even though direct protein-mRNA interaction was not demonstrated. In addition, this line of research does not provide a clear and identifiable pharmacological target with therapeutic potential.

## Summary and Conclusions

Since the discovery of the FMR1 mutation as the cause of FXS, many hypotheses have been proposed on how lack of FMRP results in dysfunctional synaptic activity. In this review, I tried to summarize most of these hypotheses, focusing predominantly on evidence obtained from the *Fmr1* KO mouse and, more recently, from human-based models, especially hPSCs.

Given the symptoms of FXS and ASDs, rationally-built hypotheses were put to the test, resulting in outstanding scientific discoveries. The “mGluR theory of FXS” proposes that lack of FMRP leads to an increase in mGluRI-dependent LTD, resulting in reduced LTP, therefore explaining the cognitive impairment and intellectual disability. The “GABAergic theory of FXS” proposes that lack of FMRP leads to reduced GABA-mediated inhibition, explaining neuronal hyperexcitation, behavioral hyperactivity and epilepsy in FXS and ASDs. Newer studies suggest that loss of FMRP affects the intrinsic properties of the neuron itself, resulting in abnormal ion channel activity and firing pattern, decreased neurotransmitter release and overall reduced synaptogenesis, which can explain many of the symptoms characterizing FXS, especially from a developmental perspective. Many of these studies have also provided the clinical world with accessible targets for pharmacological treatment: from neurotransmitter receptors, through ion channels, to cytosolic enzymes. However, so far, none of these hypotheses has been shown as definitive, or fully elucidated from a mechanistic point of view; nor as the source of a successful treatment or cure for FXS and ASDs in clinical trials.

The criticism of current FXS hypotheses in this review does not call into question the quality and the value of the research done and the data obtained, but the relevance of the model employed, in each specific case. Extrapolating from mouse to human, or from cell cultures to whole organisms, is a complex issue, for which definitive standards do not necessarily apply. For many years, it was hard to compare the results from mice to anything else. Today, hPSCs, and other human-based models, can be used to answer basic scientific questions, as well as to find molecular correlates with mouse data, increasing the relevance of the research to accelerate the finding of a suitable treatment for FXS and ASDs.

## Author Contributions

The author confirms being the sole contributor of this work and has approved it for publication.

## Conflict of Interest Statement

The author declares that the research was conducted in the absence of any commercial or financial relationships that could be construed as a potential conflict of interest.

## Abbreviations

AMPA: α-amino-3-hydroxy-5-methyl-4-isoxazolepropionic acid
aNSCs: adult neural stem cells
ASDs: autism-spectrum disorders
BDNF: brain-derived neurotrophic factor
CNS: central nervous system
cAMP: cyclic adenosine monophosphate
GABA: gamma-(γ)-aminobutyric acid
GABA_A_: ionotropic GABA receptors
GABA_B_: metabotropic GABA receptors
GluRs: glutamate receptors
iGluRs: ionotropic glutamate receptors
mGluRs: metabotropic glutamate receptors
GSK3β: glycogen synthase kinase 3 beta
FMR1: human fragile X mental retardation 1 (gene)
*Fmr1*: mouse fragile X mental retardation 1 (gene)
FMRP: FMR1/*Fmr1* protein
hPSCs: human pluripotent stem cells
hESCs: human embryonic stem cells
hiPSCs: human induced-pluripotent stem cells
KO: knock-out
LTD: long-term depression
LTP: long-term potentiation
NMDA: N-methyl-D-aspartate
TFs: transcription factors
UTR: untranslated region
WT: wild-type.
